# Analysis of clinical trials on drugs targeting dopamine receptors: a scoping review

**DOI:** 10.1007/s00210-025-04710-x

**Published:** 2025-10-17

**Authors:** Thomas Rajan, Roland Seifert

**Affiliations:** Institute of Pharmacology Hannover Medical School, 30625 Hannover, Germany

**Keywords:** Dopamine, Dopamine receptors, Parkinson, Schizophrenia

## Abstract

Dopamine receptors are G protein-coupled transmembrane proteins that mediate dopamine-dependent neurotransmission in the central nervous system via intracellular signaling cascades. Through their involvement in motor and cognitive processes, they are involved in various neurological diseases and their symptoms, including Parkinson’s disease and schizophrenia. The pharmacological modification or antagonization of dopamine receptors is therefore highly relevant. Dopamine receptors have been studied across diverse conditions, yet the resulting clinical evidence is scattered and highly heterogeneous, with no structured synthesis to date. This fragmentation limits the ability to evaluate the broader clinical relevance of dopamine receptor modulation, and a synthesis of these heterogenous trials represents a research gap. By systematically mapping and summarizing available trials, this review provides an integrated overview and highlights areas where further research is needed. The aim of this scoping review was to analyze current and completed clinical studies on dopamine receptors. Because the field of dopamine receptor–targeted therapies is broad, complex, and not yet systematically mapped, a scoping review offers the methodological flexibility to capture this diversity and synthesize current knowledge. ClinicalTrials.gov was searched for trials on dopamine receptors. Studies with a focus on any dopamine receptor were included, while studies employing dopamine receptors and related medications as comparators were excluded. Search results were evaluated based on the condition, the type of intervention, the study location, the sponsor, and the outcome. Two hundred forty-five. trials on dopamine receptors were identified. One hundred ninety-two (78.4%) trials were interventional trials and 53 (21.6%) were observational studies. Ten trials were terminated before completion. More than one-third of the studies (38.3%) were conducted in the USA. Most trials (*n* = 151) were sponsored by sponsors other than industrial or governmental entities, primarily by clinics and medical centers of universities. The most frequent indication was schizophrenia (*n* = 20, 8.0%), followed by Parkinson’s disease (*n* = 18, 7.2%), tobacco abuse (*n* = 8, 3.2%), alcoholism (*n* = 7, 2.8%), and other substance abuse (*n* = 7, 2.8%). Thirty-eight trials (15.5%) were classified as “Has results,” while for 207 trials (84.5%), no results were available. Of the trials with results, 17 had publications in which the results were reported, with 20 studies published in total. While numerous trials have been conducted on dopamine receptors, the outcome from these studies in terms of scientific publications is low. The reasons for this low output must be investigated in future studies.

**Supplementary Information:**

The online version contains supplementary material available at 10.1007/s00210-025-04710-x.

## Intoduction

### Rationale

Dopamine belongs to the monoamine catecholamines and is the precursor of the neurotransmitters norepinephrine and epinephrine (Nagatsu et al. 1964; Fernstrom and Fernstrom 2007). Dopamine acts as a neurotransmitter and is involved in signaling processes in the central nervous system and peripheral organs (Klein et al. 2019). As part of the dopaminergic system, dopamine plays a role in motor control, cognitive function, attention, arousal, motivation, reinforcement, and hormone regulation (Li et al. 2006; Bromberg-Martin et al. 2010; Klein et al. 2019). It mediates these pathways by binding to dopamine receptors, thereby initiating intracellular signaling cascades.

Dopamine receptors are G protein-coupled receptors (GPCR) that initiate intracellular signaling cascades upon dopamine binding. The therapeutic targeting of dopamine systems has a long history spanning over a century. Dopamine was first synthesized in 1911 and later identified as a neurotransmitter in the human brain during the 1950 s, shifting its status from a biochemical precursor to an active signaling molecule (Ehringer and Hornykiewicz 1960). The discovery that dopamine depletion produced Parkinsonian features in animals, and that these could be ameliorated with L-DOPA, provided the foundation for the introduction of L-DOPA therapy for Parkinson’s disease in the late 1960 s and early 1970 s (Cotzias et al. 1969). Around the same time, the clinical use of antipsychotic drugs such as chlorpromazine supported the dopamine hypothesis of psychosis, as their therapeutic effects were linked to antagonism at dopamine D₂ receptors (Seeman 2021). Subsequent decades saw the development of dopamine agonists, beginning with ergot derivatives such as bromocriptine and apomorphine, and later non-ergot drugs including pramipexole and ropinirole, which remain widely used in the management of Parkinson’s disease (Zhang and Tan 2016).

In addition to the cAMP-PKA axis, dopamine receptors may also activate the G protein α-subunit Gαq, which regulates the activity of phospholipase C, the production of inositol triphosphate and diacylglycerol, resulting in an intracellular increase in calcium levels and activation of protein kinase C (Sahu et al. 2009). Moreover, GPCR can activate extracellular-signal-regulated kinases (ERK)/mitogen-activated protein kinases (MAPK), which are involved in synaptic plasticity, cell death mechanisms, and development (Chang and Karin 2001).

Due to the broad expression of dopamine receptor subtypes in the central nervous system and peripheral tissues, these receptors have been implicated in both neurodegenerative and neuropsychiatric disorders (Zhuang et al. 2021).

In Parkinson’s disease, the dopamine pathways affected during the early disease stage are those involved in voluntary movement, resulting in motor dysfunctions including tremor, impaired balance, bradykinesia, and rigidity (Dunnett and Björklund, 1999). During the later stages, other dopamine pathways are affected, and their impairment has an impact on cognition, emotion, motivation, sensory processing, memory, and learning (de la Fuente-Fernández 2013; Narayanan et al. 2013). Dopamine signaling affects synaptic plasticity, and thereby, impaired dopamine signaling may result in different neuropsychiatric disorders, such as schizophrenia (Madadi Asl et al. 2019). Impairment of neuronal circuits and alterations in dopaminergic neurons (Guillin et al. 2007; Wang et al. 2017) and a high density of D_2_ receptors in the brain of schizophrenic patients (Frankle and Laruelle 2002) have been observed. Other disorders with dopamine involvement include attention-deficit/hyperactivity disorder (ADHD) (del Campo et al. 2013), Huntington’s disease (Cepeda et al. 2014; Koch and Raymond 2019), and certain cancers (Grant et al. 2022).

New medicinal products targeting the dopamine receptor are therefore developed as therapeutic options for patients suffering from these diseases.

### Objectives

Most clinical trials are registered in an online database of the US National Library of Medicine, ClinicalTrials.gov, with information on the target condition or disease, the target molecule, the intervention or treatment that is conducted in the trial, location where the study is conducted, the status of the study and details on the inclusion and exclusion criteria, study design, participants, sponsors, and publications resulting from the study.

The objective of this scoping review is to analyze currently ongoing and completed clinical trials on dopamine receptors by synthesizing relevant information on ClinicalTrial.gov.

## Methods

### Information sources and search

This scoping review was conducted according to the PRISMA scoping review guidelines (Tricco et al. 2018) (see checklist in supplementary Table S12). A systematic search for clinical trials on the dopamine receptor was conducted on the website ClinicalTrials.gov that serves as a register for clinical trials and is maintained by the National Library of Medicine of the National Center for Biotechnology Information. Studies with a focus on any dopamine receptor were included, while studies employing dopamine receptors and related medications as comparators were excluded. Data was extracted and evaluated for all trials and for subgroups by study type and publication status. The search protocol was not registered.

The search was conducted in July 2023 by using “dopamine receptors” as the search term.

### Data charting, data items, and synthesis of results

The following details on the clinical trials investigating different aspects of dopamine receptors were extracted:

- Study ID
- Study title
- Study status
- Study type (interventional or observational)
- Study location
- Availability of results (no results available vs. has results)
- Study condition
- Study intervention type (drug, radiation, dietary supplement, procedure, behavioral, diagnostic test, device, genetic, other)
- Study intervention element (name of interventional compound)
- Study outcome measures
- Study sponsors (lead and collaborators)
- Gender of included participants
- Minimum and maximum age of included participants
- Age group of included participants (child or adult)
- Study phase
- Study enrollment
- Study start and end date
- Study URL

**Fig. 1:**
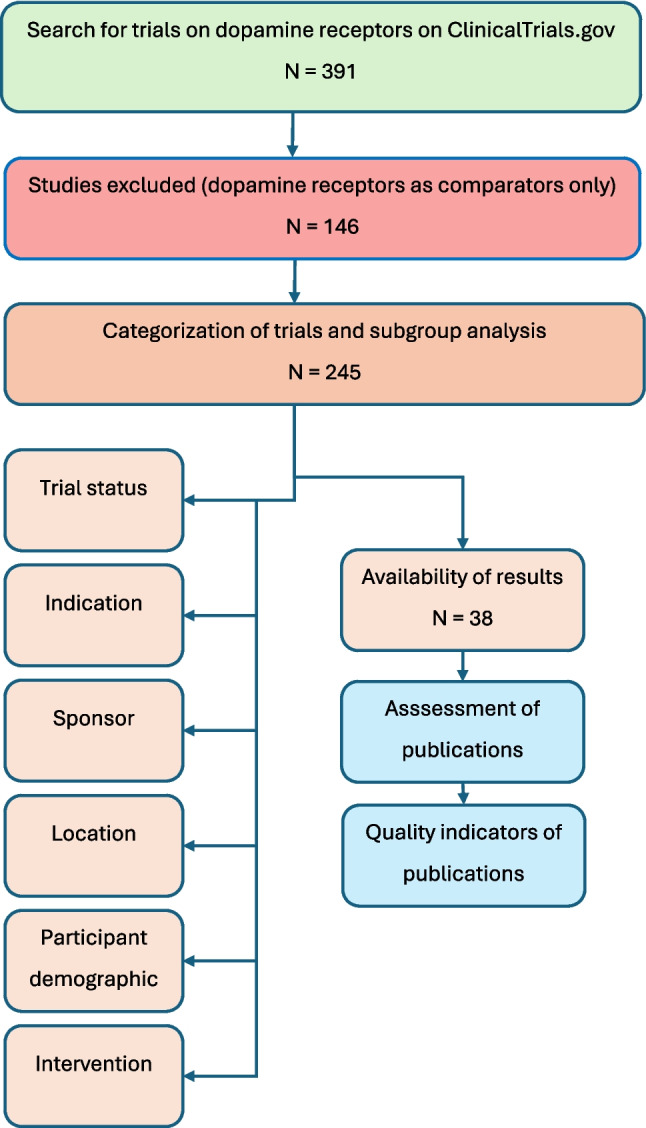
Process of clinical trial identification and evaluation

Data was compiled based on the extracted parameters. First, an overview of all identified trials on dopamine receptors was provided by summarizing the study type, phase, sponsors, conditions, interventions, and outcome measures (Fig. 1).

### Critical appraisal of individual source of evidence

In order to critically evaluate the outcome of the identified studies, the studies were separately assessed based on their status, with subgroups of completed and terminated studies and a separate analysis of those trials with published results. A quality assessment of the published trials was conducted to determine the scientific value of such trials.

Green, search stage; orange, data acquisition and synthesis; blue, outcome and quality assessment.

## Results and discussion

### Overview of clinical trials on dopamine receptors

**Fig. 2:**
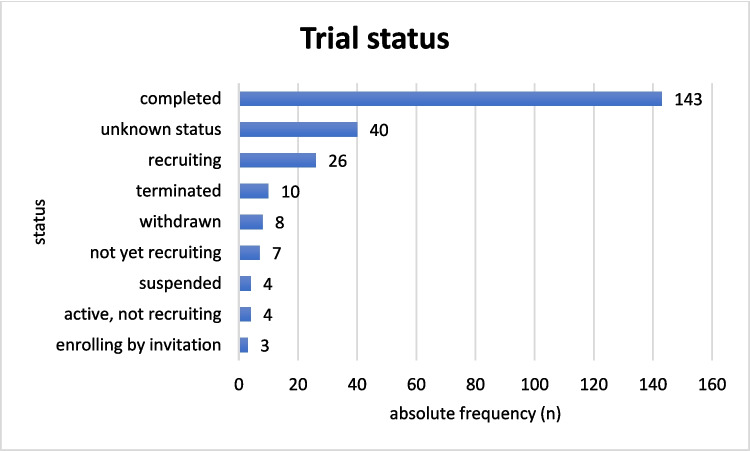
Status of the 245 identified trials on dopamine receptors

In total, 245 studies were identified in ClinicalTrials.gov using “dopamine receptor” as the search term (Fig. 1). Of these, 38 trials (15.5%) were classified as “Has results,” while for 207 trials (84.5%), no results were available. In terms of the recruitment status, most trials (*n* = 143, 58.4%) had been completed, 26 trials (10.6%) were actively recruiting, 10 trials (4.1%) had been terminated, 8 trials (3.3%) had been withdrawn, 7 trials (2.9%) were not yet recruiting, 4 trials (1.6%) had been suspended, 4 trials (1.6%) were active but not recruiting, and 3 trials (1.2%) were enrolling by invitation (Fig. 2).

**Fig. 3:**
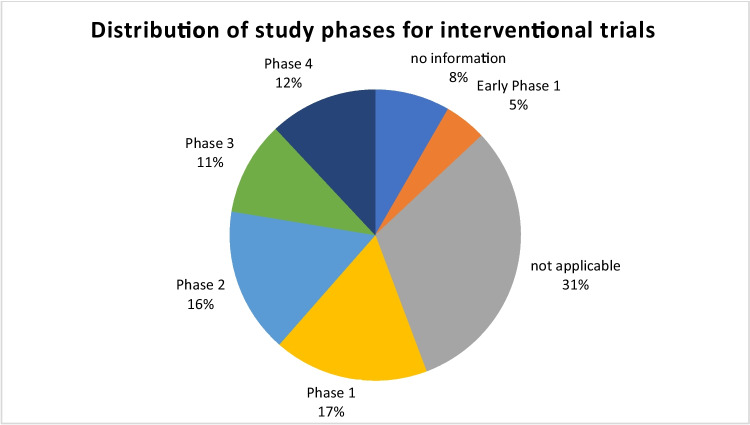
Distribution of study phases for 192 interventional trials on dopamine receptors

Of the 245 identified trials, 192 (78.4%) were interventional trials and 53 (21.6%) were observational studies. Of the 192 interventional trials, the study phase was declared as “Early Phase 1” for 9 trials, “Phase 1” for 33 trials, “Phase 2” for 31 trials, “Phase 3” for 20 trials, and “Phase 4” for 23 trials (Fig. 3). For 60 trials, the parameter study phase was deemed “not applicable”, and 16 trials did not list any information on the study phase.

### Locations

**Fig. 4:**
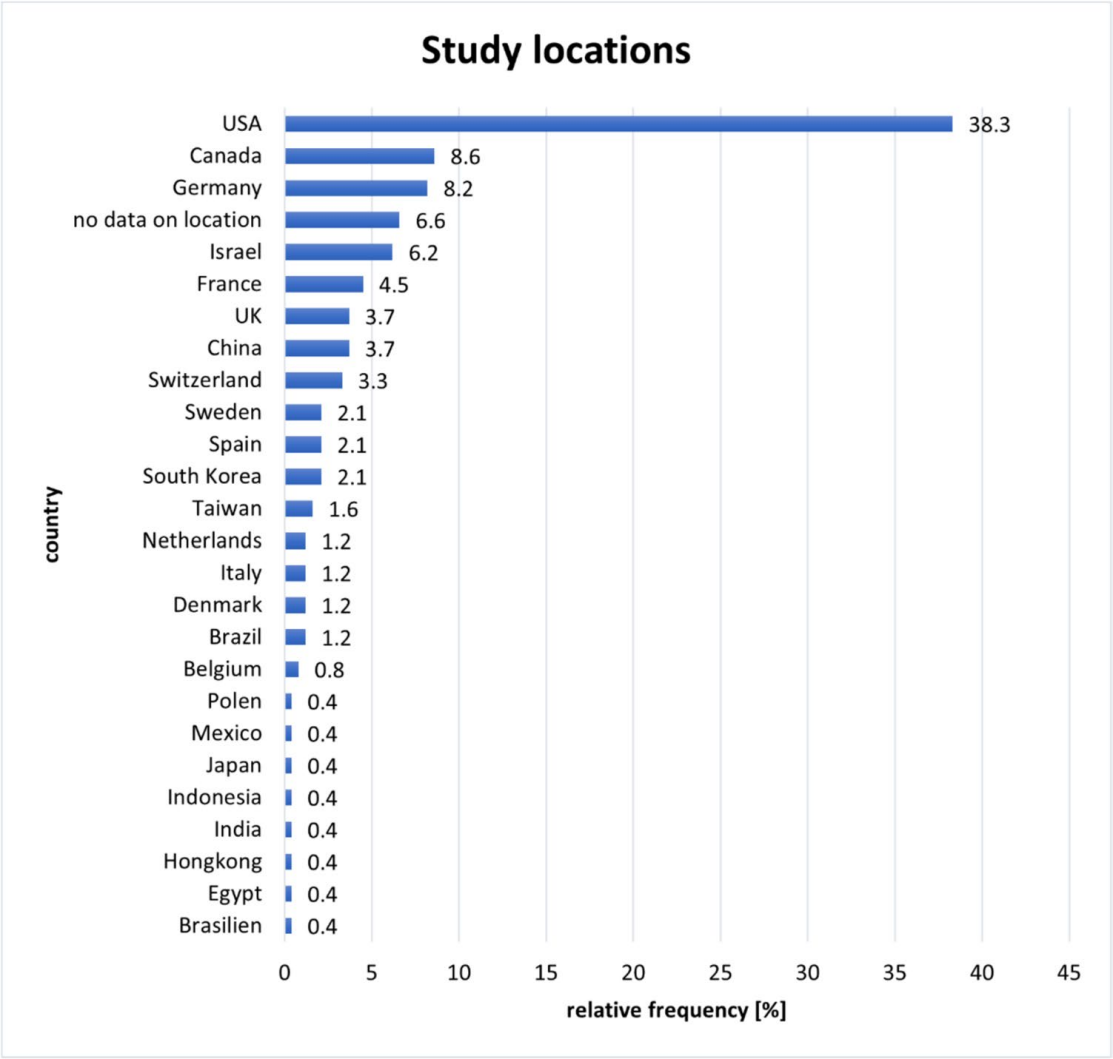
Locations of the studies on dopamine receptors

More than one-third of the studies (38.3%) were conducted in the USA. Other countries with multiple studies included Canada (*n* = 21, 8.6%), Germany (*n* = 20, 8.2%), Israel (*n* = 16, 6.6%), France (*n* = 11, 4.5%), China (*n* = 9, 3.7%), the UK (*n* = 9, 3.7%), and Switzerland (*n* = 8, 3.3%, Fig. 4).

### Sponsors

**Fig. 5:**
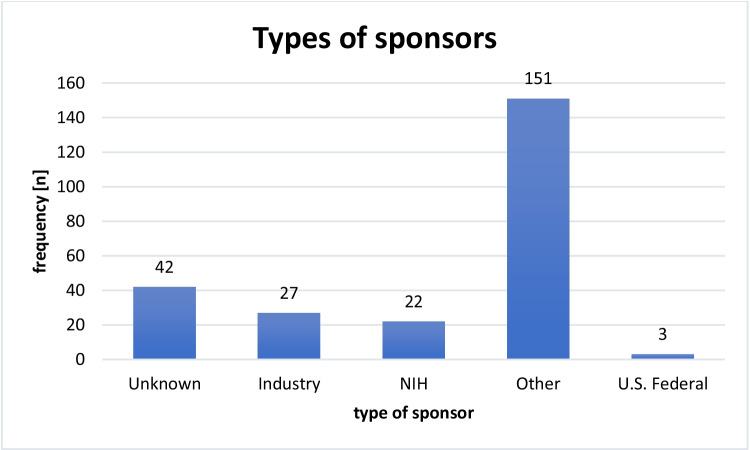
Types of sponsors of the trials by frequency

Figure 5 shows the types of sponsors of the trials on dopamine receptors. Most trials (*n* = 151) were sponsored by sponsors other than industrial or governmental entities. These included primarily clinics and medical centers of universities. The NIH sponsored 22 of the trials, the U.S. Federal Government sponsored three of the trials, and 27 trials had industrial sponsors.

In the category “other” type of sponsor, most sponsors (*n* = 47, 31.1%) were universities, followed by university hospitals (*n* = 44, 29%), hospitals and medical centers (*n* = 31, 20.5%), and health institutes (*n* = 29, 19.2%). In terms of the locations of sponsors in the category “others”, most were located in the USA (*n* = 35, 23.2%), followed by Germany (*n* = 16, 10.6%), Canada (*n* = 15, 9.9%), Israel (*n* = 15, 9.9%), France (*n* = 11, 7.3%), and China (*n* = 9, 6%, Figure S1).

### Participants

Most studies included both genders (*n* = 202, 82.4%), while 16 trials (6.5%) exclusively recruited female participants and 26 trials (10.6%) recruited only male participants. For one study (0.4%), no information was provided on the gender or genders included.

In terms of the age of the study participants, 115 trials (46.9%) listed “adult” as the age group, while one trial (0.4%) declared the age group as “older adult” and 3 trials (1.2%) stated “child” as the age group. For 126 trials (51.4%), no age group was given. For 238 trials, the minimum age of the included participants was given, and 199 trials listed a maximum age. The distribution of the minimum and maximum age of the participants is shown in the waterfall plots of Figures S2 and S3.

### Interventions

**Fig. 6:**
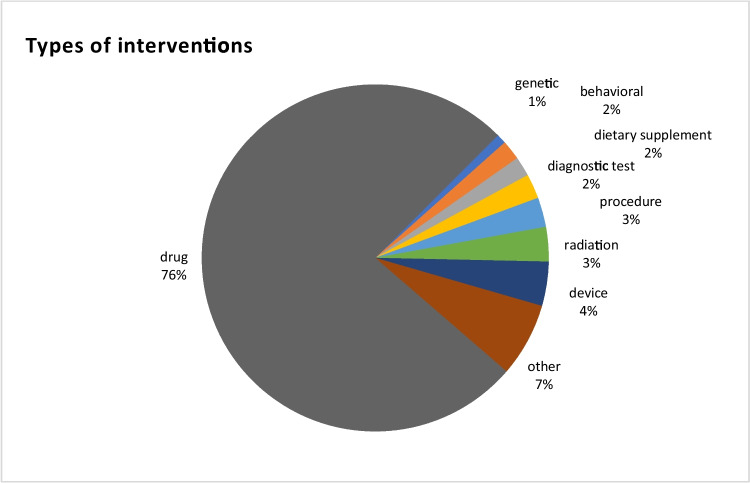
Types of intervention in 218 studies listing an intervention

Despite 53 trials listed as observational studies, an intervention was listed for 26 of these studies, for a total number of trials that listed an intervention amounting to 218. The most common type of intervention was “drug” (*n* = 166, Table S1). Other interventions included “device” (*n* = 9, Table S6), “radiation” (*n* = 7, Table S2), “procedure” (*n* = 6, Table S7), “diagnostic test” (*n* = 5, Table S4), “behavioral” (*n* = 4, Table S8), “dietary supplement” (*n* = 4, Table S5), and “genetic” (*n* = 2, Table S3) (Fig. 6). For 15 studies, the intervention was described as “other” (Table S9). Figure S7 shows the time trends in the type of intervention.

### Duration

The average duration of trials on dopamine receptors, calculated from start date to completion date, amounted to 3.2 ± 3.1 years (minimum, 0.1 years; maximum, 34 years).

Most studies using a drug as an intervention lasted 2 years (*n* = 40), 29 trials lasted 1 year, and 23 trials lasted 3 years (Figure S4).

### Indications

**Fig. 7:**
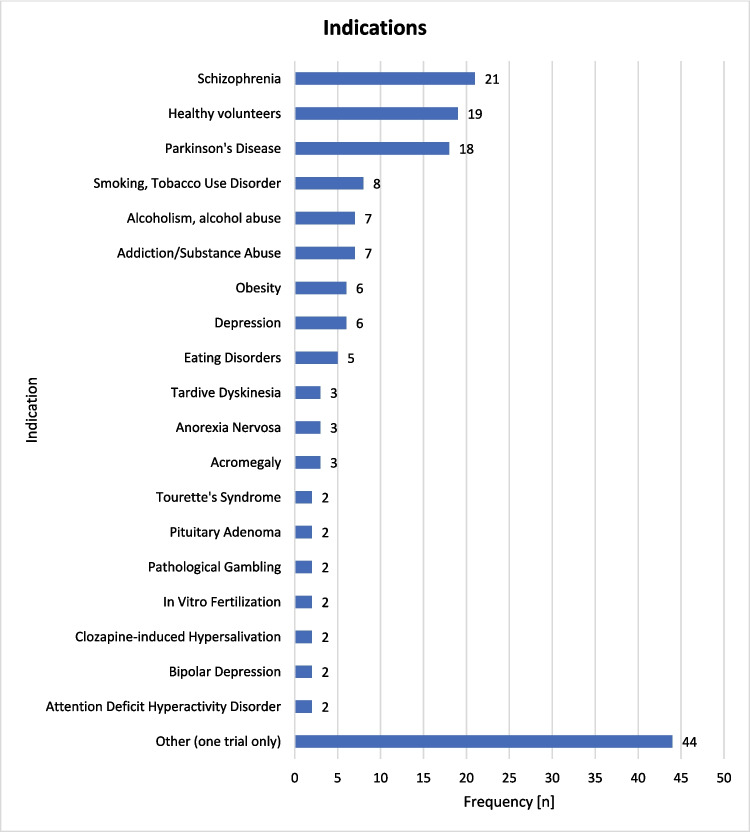
Indications of the identified trials on dopamine receptors

The indications of the trials on dopamine receptors are shown in Fig. 7. The most frequent indication was schizophrenia (*n* = 20, 8.0%), followed by Parkinson’s disease (*n* = 18, 7.2%), tobacco abuse (*n* = 8, 3.2%), alcoholism (*n* = 7, 2.8%), other substance abuse (*n* = 7, 2.8%), obesity (*n* = 6, 2.4%), depression (*n* = 6, 2.4%), and eating disorders (*n* = 5, 2.0%). Three studies each (1.2%) focused on tardive dyskinesia and acromegaly, while two studies each (0.8%) investigated Tourette’s syndrome, pituitary adenoma, pathological gambling, in vitro fertilization, clozapine-induced hypersalivation, bipolar disorder, and attention-deficit/hyperactivity disorder.

Indications that were only investigated in a single trial (summarized as “other” in Fig. 7) are listed in Table S10. Figure S8 shows the time trends by indication over time.

### Terminated studies and reasons for termination

Ten trials were terminated before completion. Of these, three focused on substance abuse (cocaine dependence, alcohol addiction, and nicotine dependence), one focused on traumatic brain injury, one on multiple myeloma, and one on 22q11.2 deletion syndrome. For three studies, no information on the conditions investigated was provided. Seven of the terminated studies employed an interventional drug, including methylphenidate (*n* = 2), paliperidone (*n* = 1), domperidone (*n* = 1), risperdal (*n* = 1), ONC201 (*n* = 1), and mifepristone (*n* = 1). One study (NCT03854942) was a placebo trial. One of the terminated trials was funded by the National Institutes of Health (NCT00951314), one by an industrial sponsor (NCT00934635), and seven by a non-industrial, non-governmental sponsor such as a clinic or university. Eight of the terminated trials were interventional and two were observational studies. The enrollment of terminated studies ranged from *n* = 1 to *n* = 43.

### Completed studies and outcome

**Fig. 8:**
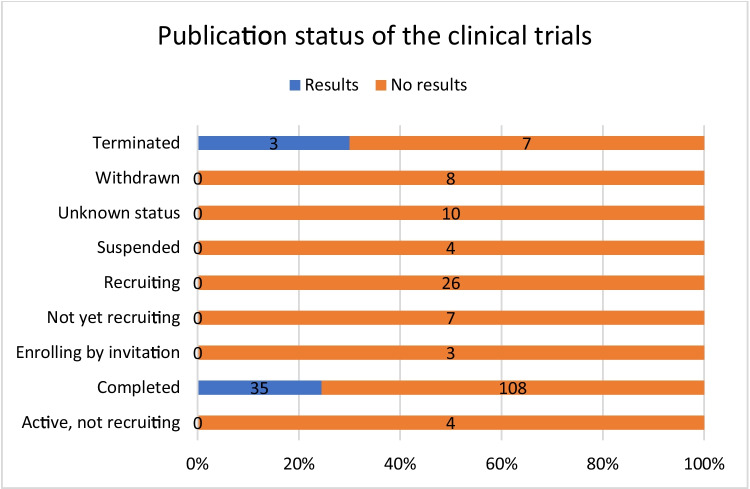
Number of studies with published results

In total, 143 studies were completed. Only completed (*n* = 35 of 143) and terminated (*n* = 3 of 10) studies had published results (Fig. 8).

In terms of the study phases of those trials with published results, eight studies were Phase 1 trials, eight studies were Phase 2 trials, seven studies were Phase 3 trials, and four studies were Phase 4 trials. For four studies, the type of study was unknown, and for five studies, the study phase was not applicable. Two of the 38 studies with published results were observational studies, and 36 were interventional studies. The age of the included patients ranged from 6 to 85 years.

In terms of the sponsorship of studies with published results, most trials (32%) were sponsored by an industrial sponsor, 5% by the National Institutes of Health, and 3% by the U.S. Federal Government (Figure S5). For 21% of the trials, the sponsor was unknown, and 39% of the trials were sponsored by a sponsor other than an industrial sponsor, such as hospitals or university medical centers.

**Fig. 9:**
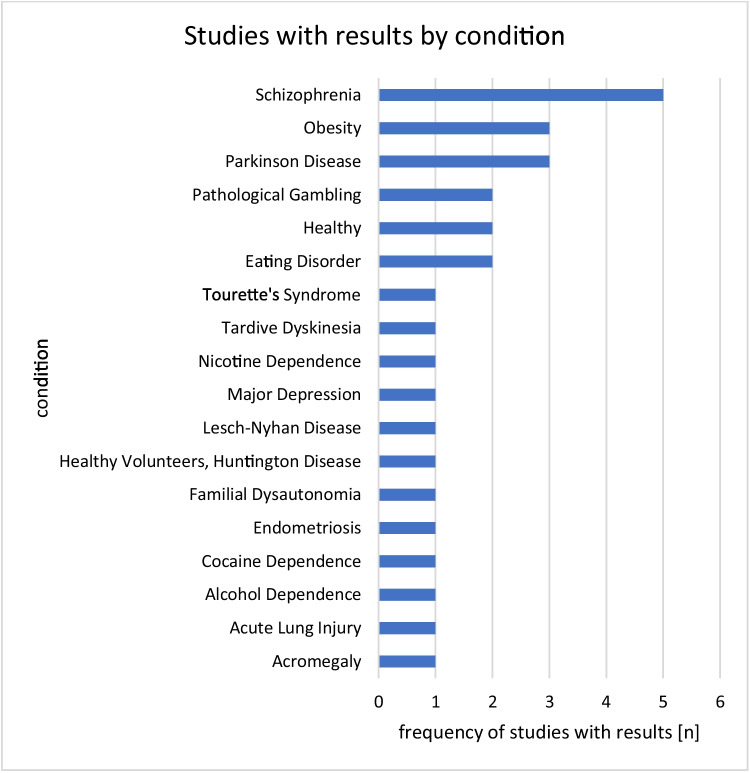
Frequency of studies with published results by condition

Figure 9 shows the frequency of studies with results by the indication. Most studies with published results (*n* = 5) were trials on schizophrenia, followed by trials on obesity (*n* = 3), Parkinson’s disease (*n* = 3), pathological gambling (*n* = 2), eating disorders (*n* = 2), and studies with healthy volunteers (*n* = 2). One trial each focusing on Tourette’s syndrome (NCT01244633), tardive dyskinesia (NCT02291861), nicotine dependence (NCT02348385), major depression (NCT00086307), Lesch-Nyhan disease (NCT01065558), Huntington’s disease (NCT03019289), familial dysautonomia (NCT01212484), endometriosis (NCT02542410), cocaine dependence (NCT02152670), alcohol dependence (NCT01657760), acute lung injury (NCT03317431), and acromegaly (NCT01278342) had published results. Ten studies with published results did not state a condition. Of the 38 studies with published results, 36 studies conducted a drug intervention, one study employed radiation as the intervention, and another study employed mechanical ventilation as the intervention.

In Table S11, the drugs used as interventional drugs in 36 of the trials with results are listed. Three studies with results used ecopipam as the interventional drug, two studies used methylphenidate, and all other listed drugs were employed in a single trial with results.

### Trends over time

**Fig. 10:**
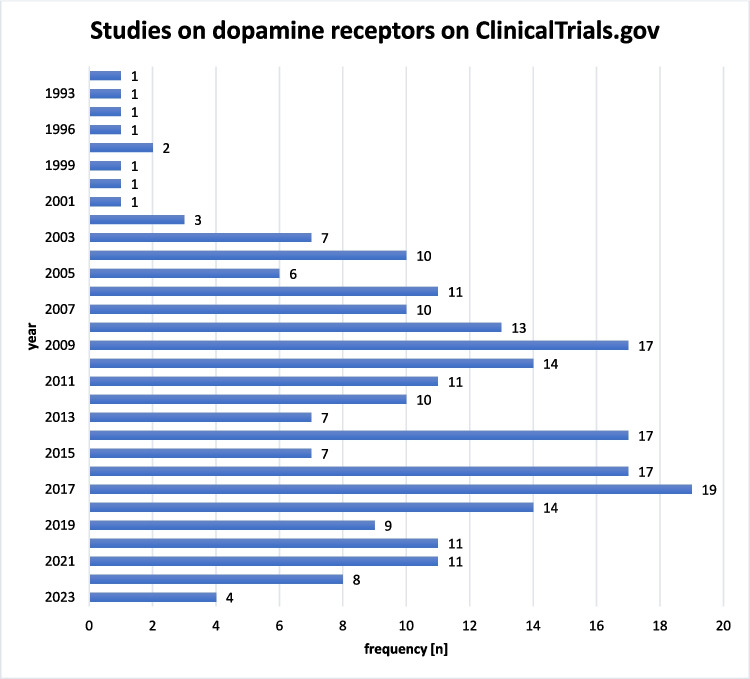
Studies on dopamine receptors on ClinicalTrials.gov by year

The number of trials on ClinicalTrials.gov was assessed by the year it was first registered. As shown in Fig. 10, the number of registered clinical trials on dopamine receptors increased from 2003, with at least six trials registered each year until the year 2022. Most trials were registered in 2017 (*n* = 19).

Parallel to the assessment of trial registrations on ClinicalTrials.gov, the number of publications on dopamine receptors increased in the early 2000 s, with the highest number of studies published in 2013 (*n* = 1824, Fig. 11). After 2013, the number of studies published per year decreased, with the lowest number of studies listed in 2022 (*n* = 1108).

**Fig. 11:**
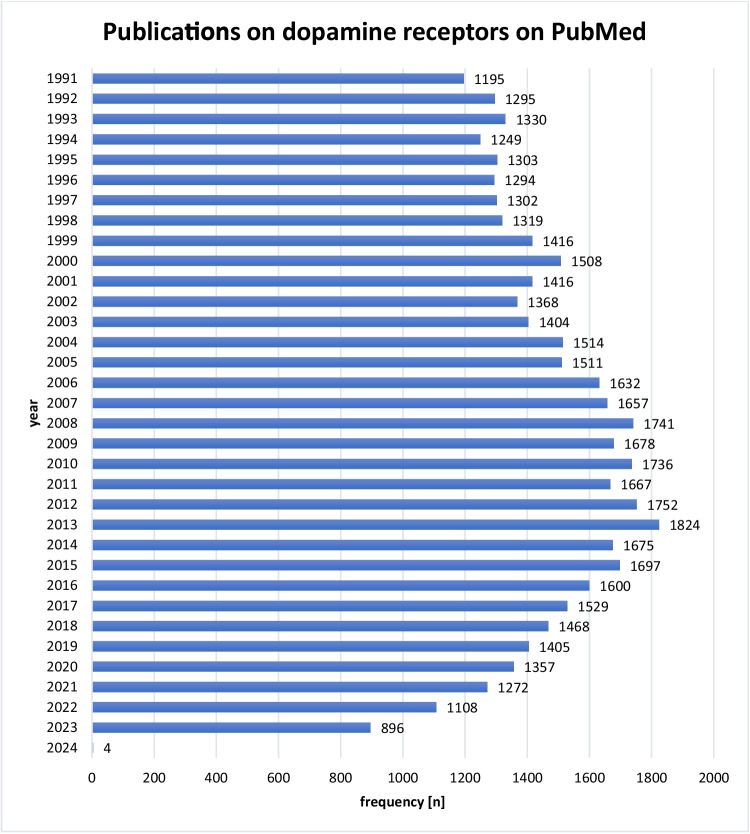
Publications on dopamine receptors on PubMed by year

**Fig. 12:**
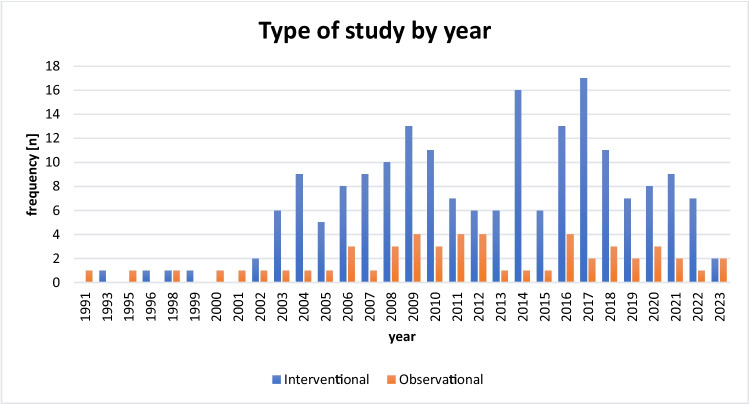
Study type over time

While the number of interventional studies increased over time, the number of observational studies remained comparatively stable (Fig. 12).

While the number of studies without results remained comparatively stable over time, the number of trials with published results decreased since 2014 (Figure S6). The types of interventions, stratified by interventions with drugs and others, are shown in Figure S7 as a yearly trend. Both drug interventions and other interventions were employed with similar frequency over the years. Figure S8 shows the studies on dopamine receptors by indication over time.

### Publications with results of clinical trials on dopamine receptors

**Fig. 13:**
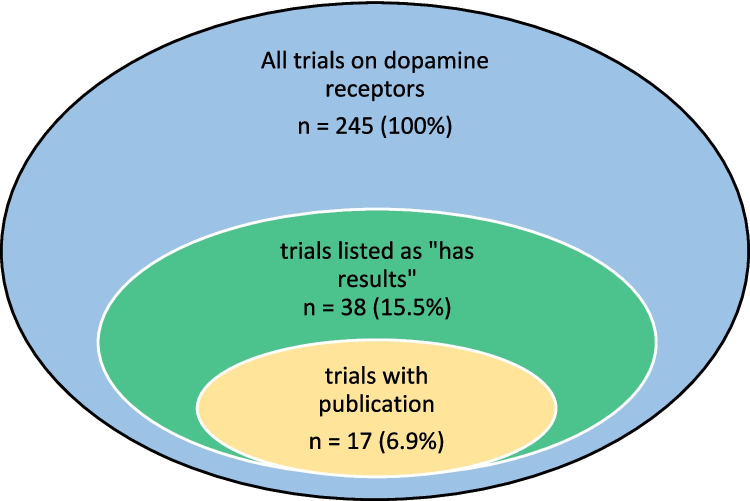
Proportion of clinical trials on dopamine receptors with reported results (“has results”) and published results (“publications about study results”)

In order to determine how many clinical trials on dopamine receptors actually resulted in a publication, the list of identified clinical trials was filtered in the database by the parameter “has results.” According to this search, 38 of the 245 trials (15.5%) were identified (Table S11). Figure S5 shows the distribution of sponsorship of these trials with published results, and Figure S6 shows the frequency of published results over time. For each of these trials, the ClinicalTrials.gov entry was accessed, and publications listed for each trial under the icon “publications” were checked. Under this icon, publications that specifically report results obtained from the respective trial are referenced (“publications about study results” and “PubMed publications”), but also “general publications” that may have been uploaded or referenced by the study lead. Such general publications are previous studies with relevance to the clinical trial that have been published before the trial, but do not report data from the current trial. Only those publications with study results and PubMed publications listing the ClinicalTrials.gov identifier of the study were evaluated in the analysis. Moreover, a hand search was conducted in PubMed using the ClinicalTrials.gov identifier as a search term. In applying this search strategy, 20 publications reporting the results from one of the trials on dopamine receptors described in this review were identified. For one trial (NCT00802204), four publications were identified, and for all other trials, only one publication was identified. Therefore, 17 trials of the 245 identified trials on dopamine receptors (6.9%) resulted in one or more publications (Fig. 13).

The 20 identified publications and the trial for which they report the results are listed in Table 1.. All of the studies for which results were reported in these publications had been completed.

**Table 1.** Publications resulting from clinical trials on dopamine receptors

#	Trial ID	Reference	Publication title	Condition	Intervention
1	NCT00592943	Spencer et al. (2010)	A positron emission tomography study examining the dopaminergic activity of armodafinil in adults using [11C] altropane and [11C] raclopride	Healthy volunteers	Drug (armodafinil)
2	NCT03019289	Grachev et al. (2021)	Sigma-1 and dopamine D_2_/D_3_ receptor occupancy of pridopidine in healthy volunteers and patients with Huntington disease: a [18F] fluspidine and [18F] fallypride PET study	Healthy volunteers	Drug (pridopidine)
3	NCT03544229	Yamaguchi et al. (2022)	Evaluating the safety, tolerability, and disposition of trazpiroben, a D_2_/D_3_ receptor antagonist: Phase I single and multiple-ascending dose studies in healthy Japanese participants	Healthy volunteers	Drug (TAK-906 maleate)
4	NCT00939523	Cooper et al. (2021)	EGFR/ErbB2-targeting lapatinib therapy for aggressive prolactinomas	Prolactinoma	Drug (lapatinib)
5	NCT01854944	Girgis et al. (2020)	A positron emission tomography occupancy study of brexpiprazole at dopamine D_2_ and D_3_ and serotonin 5-HT1A and 5-HT2A receptors, and serotonin reuptake transporters in subjects with schizophrenia	Schizophrenia	Drug (brexpi-prazole)
6	NCT00000371	Goff et al. (2005)	A 6-month, placebo-controlled trial of D-cycloserine co-administered with conventional antipsychotics in schizophrenia patients	Schizo-phrenia	Drug (D-cycloserine)
7	NCT01878006	Spagnolo et al. (2019)	Striatal dopamine release in response to morphine: a [11C]-raclopride positron emission tomography study in healthy men	Addiction	Drug (morphine)
8	NCT01657760	Zorick et al. (2022)	The effects of citalopram and thalamic dopamine D_2_/_3_ receptor availability on decision-making and loss aversion in alcohol dependence	Substance abuse/addiction	Drug (citalopram)
9	NCT02203786	Zack et al. (2023)	Priming effects of a slot machine game and amphetamine on probabilistic risk-taking in people with gambling disorder and healthy controls	Substance abuse/addiction	Drug (haloperidol)
10	NCT01215357	Grant et al. (2014)	A single-blind study of “as-needed” ecopipam for gambling disorder	Substance abuse/addiction	Drug (ecopipam)
11	NCT02348385	Zakiniaeiz et al. (2019)	Sex differences in amphetamine-induced dopamine release in the dorsolateral prefrontal cortex of tobacco smokers	Nicotine dependence	Drug (amphetamine)
12	NCT01723904	Kim et al. (2015)	Rotigotine transdermal system as an add-on to oral dopamine agonist in advanced Parkinson’s disease: an open-label study	Parkinson ‘s disease	Drug (rotigotine)
13	NCT01155466	Hauser et al. (2015)	Preladenant as an adjunctive therapy with levodopa in Parkinson’s disease—two randomized clinical trials and lessons learned	Parkinson ‘s disease	Drug (preladenant)
14	NCT01212484	Norcliffe-Kaufmann et al. (2013)	Hyperdopaminergic crises in familial dysautonomia—a randomized trial of carbidopa	Familial dysautonomia	Drug (carbidopa)
15	NCT02542410	DiVasta et al. (2021)	Nonhormonal therapy for endometriosis: a randomized, placebo-controlled, pilot study of cabergoline versus norethindrone acetate	Endometriosis	Drug (cabergoline)
16	NCT02291861	Anderson et al. (2017)	Deutetrabenazine for treatment of involuntary movements in patients with tardive dyskinesia (AIM-TD): a double-blind, randomized, placebo-controlled, phase 3 trial	Tardive dyskinesia	Drug (SD-809)
17	NCT00802204	Dunn et al. (2012)	Relationship of D_2_ binding potential with fasting neuroendocrine hormones and insulin sensitivity in human obesity	Obesity	Radiation (PET scan)
18		Garcia et al. (2015)	Lipoprotein profiles in class III obese Caucasian and African American women with nonalcoholic fatty Liver disease		
19		Dunn et al. (2017)	Caloric restriction-induced decreases in dopamine receptor availability are associated with leptin concentration		
20		Dunn et al. (2019)	Brief communication: β-cell function influences dopamine receptor availability		

A quality assessment of the published trial was conducted and is shown in Table 2, with factors that increase the scientific value of the paper shown in green, factors that decrease the scientific value shown in red, and factors that cannot be clearly assigned to one or another shown in yellow.

**Table 2:** Quality indicators for publications resulting from clinical trials on dopamine receptors

#	Trial ID	Reference	# of participants	Randomization	Placebo group	Masking	Ethical standards	Study type	Sponsors/competing interests	Primary outcome measures	Bias/confounding	Published in	Peer review	In favor of application	PubMed
1	NCT00592943	Spencer et al. (2010)	12	Yes	No	No	Observed	Observational study	Study was sponsored by manufacturer	Dopamine release and dopamine transporter occupancy after armodafinil administration analyzed by PET scan	Study type, sponsor	Biol Psychiatry	Yes	Yes	Yes
2	NCT03019289	Grachev et al. (2021)	14	No	No	No	Observed	Observational study	Study was sponsored by manufacturer	D2/D3 receptor occupancy after pridopidine administration analyzed by PET scan	Study type, sponsor	Eur J Nucl Med Mol Imaging	Yes	Yes	Yes
3	NCT03544229	Yamaguchi et al. (2022)	24	Yes	Yes	Yes	Observed	RCT	Study was sponsored by manufacturer	Serum prolactin after trazpiroben	Sponsor	Clin Pharmacol Drug Dev	Yes	Yes	Yes
4	NCT00939523	Cooper et al. (2021)	4	No	No	No	Observed	Observational study	None declared	Reduction in tumor dimension after lapatinib adminstration analyzed by MRI	Study type	J Clin Endocrinol Metab	Yes	Yes	Yes
5	NCT01854944	Girgis et al. ( 2020)	12	No	No	No	Observed	Observational study	Study was sponsored by manufacturer	D2/D3 receptor occupancy after brexpiprazole administration analyzed by PET scan	Study type, sponsor	Neuropsychopharmacol	Yes	Yes	Yes
6	NCT00000371	Goff et al. (2005)	26	Yes	Yes	Yes	Observed	RCT	None declared	Negative symptoms and cognitive impairment in schizophrenia after D-cycloserine administration analyzed by SANS and PANSS symptom scores	None	Psychopharmacol	Yes	No	Yes
7	NCT01878006	Spagnolo et al. (2019)	15	No	Yes	Yes	Observed	Observational study	None declared	Striatal dopamine release after intravenous infusion of morphine measured by [11C]-raclopride displacement	Study type	Biol Psychiatry	Yes	Yes	Yes
8	NCT01657760	Zorick et al. (2022)	25	No	Yes	Yes	Observed	Crossover study	None declared	Effect of citalopram on D2/D3 receptor, decision making and impulsivity (Balloon Analogue Risk Tass, Loss of Aversion Gambling Task)	Study type	Psychiatry J	Yes	Yes	Yes
9	NCT02203786	Zack et al. (2023)	60	No	Yes	No	Observed	Controlled trial	None declared	Effect of amphetamine and dopamine on gambling behavior (South Oaks Gambling Screen)	Study type	J Clin Exp Neuropsychol	Yes	Yes	Yes
10	NCT01215357	Grant et al. (2014)	28	No	Yes	Yes	Observed	Crossover study	None declared	Effect of ecopipam on gambling behavior (Yale-Brown Obsessive Compulsive Scale Modified for Pathological Gambling)	Study type	Ann Clin Psychiatry	Yes	Yes	Yes
11	NCT02348385	Zakiniaeiz et al. (2019)	49	No	No	No	Observed	Controlled trial	None declared	Cortical dopamine release after amphetamine administration, PET scans	None	Neuropsychopharmacology	Yes	Yes	Yes
12	NCT01723904	Kim et al. (2015)	90	No	No	Yes	Observed	Observational study (open label)	Study was sponsored by manufacturer	Effect of rotigotine (dopamine agonist) treatment on Clinical Global Impressions of Parkinson patients	Study type, sponsor	BMC Neurology	Yes	Yes	Yes
13	NCT01155466	Hauser et al. (2015)	1254	Yes	Yes	Yes	Observed	RCT	Study was sponsored by manufacturer	Effect of preladenant on off-time in Parkinson patients	Sponsor	JAMA Neurol	Yes	No	Yes
14	NCT01212484	Norcliffe-Kaufmann et al. (2013)	12	Yes	Yes	Yes	Observed	RCT	None declared	Antiemetic effect of carbidopa in patients with familial dysautonomia	None	Neurology	Yes	Yes	Yes
15	NCT02542410	DiVasta et al. (2021)	9	Yes	Yes	Yes	Observed	RCT	None declared	Effect of norethindrone acetate on pelvic pain in patients with endometriosis	None	Fertil Steril Rep	Yes	Yes	Yes
16	NCT02291861	Anderson et al. (2017)	813	Yes	Yes	Yes	Observed	RCT	Study was sponsored by manufacturer	Effect of deutetrabenazine on symptoms of tardive dyskinesia (Abnormal Involuntary Movement Scale)	Sponsor	Lancet Psychiatry	Yes	Yes	Yes
17	NCT00802204	Dunn et al. (2012)	22	No	No	No	Observed	Observational study	None declared	Relationship between D2 receptor, insulin sensitivity, and leptin in lean and obese women (PET scan)	Study type	Diabetes Care	Yes	N/A	Yes
18		Garcia et al. (2015)	70	No	No	No	Observed	Observational study	None declared	Lipoprotein profiles in obesity	Study type	PLOS ONE	Yes	N/A	Yes
19		Dunn et al. (2017)	15	No	No	No	Observed	Interventional study	None declared	Effect of very-low-calorie-diet on D2/D3 binding potential (PET scan)	Study type	Obesity (Silver Spring)	Yes	N/A	Yes
20		Dunn et al. (2019)	26	No	No	No	Observed	Observational study	None declared	D2/D3 binding potential in relation to insulin sensitivity (PET scan)	Study type	PLOS ONE	Yes	N/A	Yes

To provide an overview of the scientific value of the identified clinical trials, a structured assessment was applied that considered study design alongside other quality indicators. While randomized controlled trials (RCTs) are generally regarded as the highest standard for evaluating efficacy because they minimize bias and confounding, we acknowledge that Phase I and Phase I/II trials fulfill different aims—primarily the evaluation of safety, tolerability, and pharmacokinetics—and should not be judged by efficacy criteria alone. Therefore, in addition to trial design, further quality indicators were considered, such as sample size, competing interests, and potential confounding factors, which are known to influence the robustness of clinical evidence (Ioannidis 2005; Lundh et al. 2017). This approach highlighted that much of the published evidence in this field originates from early-phase or pilot studies, while simultaneously recognizing that the ultimate strength of evidence depends on a combination of study design and contextual factors rather than randomization alone.

Three trials with published results were conducted with healthy volunteers to test the efficacy of dopamine receptor drugs (Spencer et al. 2010; Grachev et al. 2021; Yamaguchi et al. 2022). These studies are important because they provide controlled evidence on how dopamine receptor modulation translates into measurable biological effects, thereby informing both mechanistic understanding and potential therapeutic applications.

Spencer et al. (2010) tested the mechanism by which armodafinil, a neuropsychiatric drug, may exhibit its effects and whether dopamine receptors are involved in this mechanism. Healthy volunteers (*n* = 12) were administered modafinil, and the binding of the drug to dopamine receptors in the brain was assessed with positron emission tomography. Armodafinil was found to bind to dopamine receptors in the striatum, and a concomitant dose-dependent reduction in extracellular dopamine levels was observed. The authors concluded that the drug indeed exerts its effects through dopamine receptors and may be employed as a treatment option for neuropsychiatric disorders. This finding directly links dopamine receptor engagement to the drug’s therapeutic potential in disorders characterized by altered dopaminergic neurotransmission.

Grachev et al. (2021) tested whether the drug pridopidine binds primarily to dopamine receptors or to sigma-1 receptors. Healthy volunteers (*n* = 15) and patients with Huntington’s disease (*n* = 3) were administered pridopidine, and the binding to sigma-1 receptors and D_2_ and D_3_ receptors was evaluated using positron emission tomography. The results demonstrated that pridopidine bound to both the dopamine and sigma-1 receptors, but at higher concentrations of the drug, only sigma-1 receptors were occupied. The authors concluded that these results have clinical implications due to the dependency of the drug’s binding behavior on the dosage. This dose-dependent receptor selectivity highlights the complexity of pharmacological interactions in the dopamine system and suggests that clinical efficacy and side-effect profiles may vary substantially depending on dosage, receptor subtype involvement, and disease context.

Yamaguchi et al. (2022) investigated the efficacy, mechanism of action, and safety of trazpiroben in healthy volunteers (*n* = 24). The volunteers were randomized into three groups with different doses of trazpiroben. Each group also contained volunteers who received a placebo. Traziproben was found to be safe, and the volunteers tolerated the drug well at all dosages. An increase in serum prolactin was observed. Because prolactin secretion is tightly regulated by dopaminergic tone via D2 receptor signaling in the pituitary, these findings provide functional confirmation of dopamine receptor involvement, offering a mechanistic biomarker of target engagement.

### Proportion of trials relative to all trials per condition

**Fig. 14:**
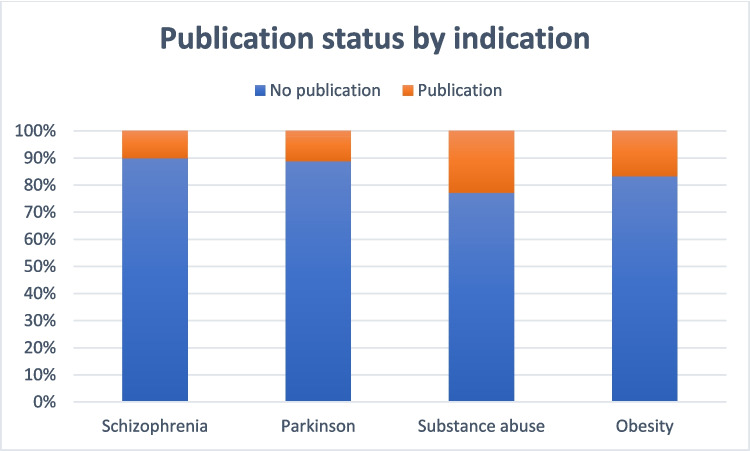
Proportion of trials with published results on dopamine receptors in schizophrenia

The proportion of studies with published results are shown for each condition with more than one trial in Fig. 14. These graphs demonstrate that only a fraction of trials on a given condition resulted in the publication of the results in peer-reviewed journals, with a publication rate ranging from 10 to 23%.

One trial with published results investigated the efficacy of a dopamine receptor drug in prolactinoma treatment (Cooper et al. 2021). In this trial, Cooper et al. (2021) tested whether a particular pathway, the ErbB signaling pathway, could be a potential target for drugs interfering with dopamine receptors. The overall goal was to evaluate whether such drugs may constitute treatment options for prolactinomas. The drug did not reduce the tumor dimensions to the extent that was initially achieved, yet three of the four patients demonstrated a stabilization of their disease. The drug was associated with high tolerability and minor to moderate side effects.

Two trials with published results focused on the applicability of dopamine receptor drugs in schizophrenia (Goff et al. 2005; Girgis et al. 2020). Girgis et al. (2020) tested the effect of the drug brexpiprazole in patients with schizophrenia (*n* = 12) on dopamine receptors and the serotonin transporter using positron emission tomography. The occupancy of dopamine receptor D_2_ was shown to be dose-dependent, while D_3_ receptors were occupied by brexipiprazole at low doses and this occupancy was not affected by higher doses of the drug.

Goff et al. (2005) evaluated the efficacy of D-cycloserine, an inhibitor of dopamine receptors D_2_ and D_3_, as a neuropsychiatric drug, in patients with schizophrenia (*n* = 55). The drug showed no efficacy in reducing the patients’ schizophrenia-related symptoms.

Five trials had published results on dopamine receptors as targets for addiction treatment (Grant et al. 2014; Spagnolo et al. 2019; Zakiniaeiz et al. 2019; Zorick et al. 2022; Zack et al. 2023). Zakiniaeiz et al. (2019) tested the correlation between amphetamine and D_2/3_ occupancy in smokers (*n* = 24) and non-smokers (*n* = 25). Participants were evaluated by positron emission tomography before and after amphetamine administration. In smokers, more D_2_ receptors were occupied than in non-smokers under amphetamine use, and dopamine release was particularly impaired by amphetamine, particularly in women.

Zorick et al. (2022) tested the effect of citalopram on dopamine receptor availability and behavioral as well as emotional aspects of participants with alcohol dependence (*n* = 12) in comparison to healthy controls (*n* = 12). The effects of citalopram were tested by behavioral tests and dopamine receptor occupancy by positron emission tomography. The occupancy of dopamine D_2_ and D_3_ receptors correlated negatively with loss aversion. No differences between alcohol-dependent participants and healthy individuals were observed in terms of delay discounting and risk taking.

Spagnolo et al. (2019) investigated the effect of a morphine injection on the dopamine response in the striatum. Healthy volunteers (*n* = 15) received morphine injections and dopamine release was assessed by positron emission tomography. A diminished release of dopamine was observed following the morphine injections, indicating that morphine interacts with dopamine synthesis or the dopamine receptors.

Grant et al. (2014) published a review article summarizing their own clinical trial and other preclinical and clinical trials on drugs targeting the dopamine receptors in cancer patients.

Zack et al. (2023) focused on people who are addicted to gambling and assessed their risk-taking behavior in correlation with amphetamine use. Participants with gambling disorder were compared to healthy controls in terms of their net monetary outcomes following amphetamine use and injection of a dopamine receptor antagonist. The results demonstrated that risky behavior increased with amphetamine use, yet this was the case across all groups with and without dopamine receptor antagonist pretreatment, and therefore, a link to dopamine receptors in the amphetamine response could not be clearly delineated.

Two trials on dopamine receptors for the treatment of Parkinson’s disease had published results (Hauser et al. 2015; Kim et al. 2015). Hauser et al. (2015) assessed whether the addition of the drugs preladenant and rasagiline to the treatment regimen of Parkinson’s patients taking levodopa could alleviate their motor symptoms between the levodopa intervals (= off time). Off time was found to be not significantly reduced by the addition of preladenant or rasagiline, indicating that the addition of these drugs does not contribute an additional benefit to levodopa.

Kim et al. (2015) treated Parkinson’s patients with rotigotine in addition to levodopa and dual dopamine receptor antagonists to determine the efficacy and safety of the drug in this patient group. Administration of the drug resulted in a reduction in the off time and improved sleep problems and activities of daily living.

One trial with published results focused on dopamine receptors as a treatment target for familial dysautonomia (Norcliffe-Kaufmann et al. 2013). Norcliffe-Kaufmann et al. (2013) evaluated whether carbidopa may exert antiemetic properties in patients with familial dysautonomia (*n* = 12) by inhibiting dopa-decarboxylase and thereby diminishing dopamine production. Carbidopa proved to be an effective antiemetic in this patient group and was well tolerated by the patients.

One trial with published results focused on dopamine receptors as a treatment target for endometriosis (DiVasta et al. 2021). DiVasta et al. (2021) assessed whether cabergoline may alleviate pain experienced by patients with endometriosis (*n* = 9). The results indicated that the drug improved pelvic pain, and that this effect was more pronounced than the pain reduction achieved by standard therapy.

One trial with published results focused on dopamine receptors as a treatment target for tardive dyskinesia (Anderson et al. 2017). Anderson et al. (2017) evaluated the efficacy and safety of deutetrabenazine, an inhibitor of dopamine receptors, for the treatment of patients with tardive dyskinesia (*n* = 222). Patients received the drug as a dose escalation and the effect on disease symptoms was tested on a standardized scale, the Abnormal Involuntary Movement Scale. Deutetrabenazine reduced tardive dyskinesia symptoms and was found to be a safe and well-tolerated drug.

The trial with the most publications investigated dopamine receptors as a treatment target for obesity (Dunn et al. 2012, 2017, 2019; Garcia et al. 2015).

In summary of the four publications, the occupancy of D_2_ in patients with obesity and diminished insulin sensitivity was evaluated by positron emission tomography and correlated with parameters that are relevant for patients with obesity and diminished insulin sensitivity, such as BMI and leptin. Both BMI and leptin correlated positively with D_2_ occupancy in the brain (Dunn et al. 2012). In another publication, occupancy of D_2_ and D_3_ was investigated in the context of the Roux-en-Y-gastric bypass, and dopamine receptor availability correlated with leptin concentrations and weight loss (Dunn et al. 2017). In a third study, the correlation between insulin sensitivity and dopamine receptor occupancy was analyzed, and the results showed a positive correlation between low insulin sensitivity and the occupancy of dopamine D_2_ and D_3_ receptors in obese patients (*n* = 18).

Our analysis shows that although dopamine receptors are deeply implicated in motor, cognitive, and neuroendocrine processes, only a minority of completed clinical trials result in published findings, with overall publication rates ranging from 10 to 23%. This scarcity of accessible data limits the ability to draw robust conclusions on how modulation of dopamine receptor signaling translates into clinical benefit across disease contexts.

The trials with available results nonetheless provide insight into the diverse roles of dopamine receptors across neurological and systemic disorders. In schizophrenia, studies on brexpiprazole and D-cycloserine underscore the centrality of D2 and D3 receptor occupancy for antipsychotic efficacy, while also illustrating the challenges of translating receptor binding into consistent symptom relief. These findings resonate with the established role of dopamine receptor dysregulation in mesolimbic and mesocortical signaling cascades, suggesting that more nuanced receptor-selective strategies may be required.

In addition, PET-based occupancy studies highlight sex- and smoking-related differences in dopamine release, as well as interactions between serotonergic agents and dopamine receptor availability. Such findings contribute to understanding the neurobiological substrates of reward and risk-taking behavior, consistent with the hypothesized role of dopamine receptor signaling in reinforcement and impulsivity pathways.

In Parkinson’s disease, adjunctive therapies targeting dopamine receptors (preladenant, rasagiline, rotigotine) produced mixed results. The lack of benefit with adenosine–dopamine interactions versus the symptomatic improvements seen with dopaminergic agonists reflects the complexity of compensating for nigrostriatal dopamine loss. This aligns with the broader challenge of fine-tuning dopamine receptor signaling in basal ganglia circuits to optimize motor and non-motor outcomes.

The smaller trials in prolactinomas, familial dysautonomia, endometriosis, and tardive dyskinesia show that dopamine receptor pharmacology extends beyond classical neuropsychiatric disorders, influencing endocrine signaling, gastrointestinal motility, pain, and involuntary movement control. These results highlight the wide-ranging physiological functions of dopamine receptor–mediated signaling cascades, but also the need for larger confirmatory studies.

Finally, the series of trials in obesity provides evidence linking D2/D3 receptor occupancy with BMI, leptin, and insulin sensitivity, directly connecting dopamine receptor availability to metabolic regulation. This reinforces the view of dopamine receptors not only as mediators of reward pathways but also as modulators of systemic energy homeostasis.

Taken together, the published clinical trials support the conceptual framework introduced in our abstract and introduction: dopamine receptor signaling cascades are central to diverse pathophysiological processes, yet their clinical exploitation remains fragmented and underreported. Bridging this gap will require more consistent publication of trial outcomes, mechanistically informed trial design, and integration of receptor occupancy data with disease-specific endpoints. By mapping both what is known and what remains absent from the literature, this review identifies critical opportunities to advance dopamine receptor research in line with the diseases where their dysfunction is most consequential.

### Limitations

This study has certain limitations. Despite comprehensive efforts to ensure the completeness of the trials included, it is possible that some relevant publications or sub-trials were inadvertently missed. The dynamic nature of clinical research means that the status of some trials may have been updated or changed during the time between data collection and analysis, potentially impacting the comprehensiveness of our review.

Moreover, dopamine receptors, which are central to this investigation, may be a component of additional studies that were not identified by our search strategy. The presence of dopamine receptors in multifactorial or broader clinical investigations could complicate their detection when using standard database queries, such as those conducted in ClinicalTrials.gov. In addition to using the term dopamine receptors, we also included broader terms such as dopamine and dopaminergic in our search on ClinicalTrials.gov. However, we found that these broader terms returned a large number of highly heterogeneous and often non-specific results, many of which did not pertain directly to our focus. Our intention in narrowing the search to “dopamine receptors” was to ensure relevance and specificity to the research question, which centers on interventions directly targeting the receptors themselves. Nonetheless, we acknowledge that the reliance on keyword-based searches, although thorough, may fail to capture all nuances of the trial landscape, particularly when dopamine receptor modulation is only a secondary or tertiary focus within complex study designs.

Additionally, the variability in the reporting practices of clinical trials poses another challenge. Some studies may not explicitly describe their focus on dopamine receptors or may use terminology that differs from established keywords, further complicating retrieval efforts. As a result, this dataset may underrepresent the totality of research involving D2 receptors, and the conclusions drawn should be interpreted with this consideration in mind. We acknowledge that ClinicalTrials.gov is not a comprehensive source and that its entries rely on investigator-reported information without independent verification. As a result, inconsistencies such as misclassification of study phase or type may occur, introducing potential bias. While we carefully screened entries, full verification of study details was not feasible. To mitigate this limitation, our analysis emphasized peer-reviewed publications and reported results rather than registry classifications alone. Because only a small proportion of the registered clinical trials resulted in published outputs, it was not possible to assess trends in study status in relation to the timing of trial initiation. We further acknowledge that ClinicalTrials.gov does not serve as a comprehensive repository of *all* clinical trials worldwide. Registration requirements vary across countries, and some studies may be recorded in other national or regional registries, which limits the completeness of our dataset.

Future research could address these limitations by employing more advanced and diverse search strategies. Furthermore, regular updates to trial status and the continuous monitoring of emerging research findings are crucial to maintaining the accuracy and relevance of systematic reviews in rapidly evolving fields. Since the initial systematic search for clinical trials was conducted, four additional studies focusing on dopamine receptors have been registered, as indicated by ClinicalTrials.gov identifiers NCT06413433, NCT06360419, NCT06300580, and NCT06261905. While these have not been included in the evaluation of the present study, the details are summarized in Table 3. None of these trials has yet resulted in publications, as all four studies are in the recruiting phase.

**Table 3:** Trials on dopamine receptors initiated since completion of the present study

NCT number	Study status	Condition	Interventions	Sponsor	Study type
NCT06413433	Recruiting	Binge-eating disorder	Solriamfetol 150 mg or 300 mg, placebo	Axsome Therapeutics, Inc	Interventional
NCT06360419	Recruiting	Major depressive disorder	Solriamfetol 300 mg, placebo	Axsome Therapeutics, Inc	Interventional
NCT06300580	Recruiting	Healthy volunteers	ABBV-932	AbbVie	Interventional
NCT06261905	Recruiting	Opioid use disorder	[11C]-PHNO| dietary supplement: calcitriol, placebo	Yale University	Interventional

This increase in ongoing trials highlights a sustained interest in clinical applications and therapeutic potential associated with dopamine receptor modulation. The initiation of these studies underscores the importance of dopamine receptors in various clinical conditions and suggests a growing recognition of their role in potential treatment pathways. On the other hand, despite longstanding interest in dopamine receptors as therapeutic targets, many clinical programs have been discontinued due to limited efficacy, safety concerns, or unfavorable risk–benefit profiles in specific indications. For example, while dopamine receptor modulators showed initial promise in substance use disorders, large-scale trials often failed to demonstrate consistent or durable clinical benefits, leading to diminished enthusiasm for this application. In psychiatric disorders, issues such as heterogeneity of patient populations, off-target effects, and intolerable side effects (e.g., extrapyramidal symptoms, metabolic disturbances) further limited success. Additionally, the pleiotropic role of dopamine across multiple neural circuits complicates selective targeting, making it difficult to achieve therapeutic effects without disrupting cognition, motivation, or reward processing. These challenges have contributed to the abandonment of many dopamine receptor–focused trials, underscoring the need for more nuanced approaches, such as circuit-specific modulation or receptor subtype–selective strategies, to realize their therapeutic potential.

## Conclusions

Numerous clinical trials have been conducted or are currently being conducted on drugs or other interventions targeting dopamine receptors. Nonetheless, out of 245 identified trials, only 38 even reported results as outcomes from the study, and only 17 trials have published results. This, in turn, means that the majority of trials do not conclude with publishable results, which raises the question of why the outcome is so low. The low number of publications resulting from lengthy and complex trials appears inefficient, as the primary goal of a clinical trial is to generate meaningful results for the tested outcome measures and serve as a basis for clinical applications. Nonetheless, in order to increase the reliability and applicability of such results in the clinical practice, a thorough evaluation and peer review, as it is conducted during the publication process, are required. Based on the results of this review, the reasons for the low publication rates remain unknown, but should be further investigated in future studies to corroborate the results and increase the output from such trials. Limitations of this study include the potential for overlooking published trials as they were searched by trial identification number and not all study investigators necessarily update their trials on ClinicalTrials.gov regularly.

## Supplementary Information

Below is the link to the electronic supplementary material. 

ESM1 (DOCX 278 KB)

## Data Availability

All source data for this work are available upon reasonable request.

## Abbreviations

ADHD: Attention-deficit/hyperactivity disorder
cAMP: Cyclic AMP
CREB: CAMP response element-binding protein
CTD: Common technical document
DARPP-32: Dopamine and cAMP-regulated phosphoprotein 32-kDa
DOPA: 3,4-Dihydroxyphenylalanine
ERK: Extracellular-signal-regulated kinase
GDP: Guanosine diphosphate
GPCR: G protein-coupled receptors
GTP: Guanosine triphosphate
LC-3: Light chain 3
MAPK: Mitogen-activated protein kinases
PKA: Protein kinase A
SLC6A3: Solute carrier 6A3
VMAT2: Vesicular monoamine transporter 2
