# Supplementary material for: Analysis of clinical trials on drugs targeting dopamine receptors: a scoping review
Source: Naunyn Schmiedebergs Arch Pharmacol. 2025 Oct 17;399(3):4169–91. doi: 10.1007/s00210-025-04710-x (PMC12935844; doi:10.1007/s00210-025-04710-x)
Supplement: Supplementary file 1 — (DOCX 278 KB) [file 210_2025_4710_MOESM1_ESM.docx]

The details of the interventions are listed in Tables S1 to S9.

**Table S1**: Description of interventions classified as “Drug”

| **StudyID** | **Type of drug** |
| --- | --- |
| NCT00062946 | (18F)fallypride |
| NCT00088517 | [11C]NNC-112 |
| NCT02020408m NCT03190954 | [11C]raclopride |
| NCT02379338 | [18F]Fluortriopride |
| NCT02815917 | [18F]FTP PET/CT |
| NCT01461083 | [18F]MPPF |
| NCT03648892 | [c11] raclopride |
| NCT01638364 | Alcoholic beverage |
| NCT04128683, NCT01154829, NCT01555814, NCT00534573 | Amisulpride |
| NCT02348385 | Amphetamine |
| NCT00472355, NCT00437177 | Apomorphine |
| NCT02979548 | Aprepitant |
| NCT01562808, NCT01082848 | Aripiprazole |
| NCT00592943 | Armodafinil |
| NCT00480246 | BL 1020 |
| NCT02233829 | Brain Dopamine Reactivity |
| NCT01854944 | Brexpiprazole |
| NCT00315081 | Bromocriptin |
| NCT00846339, NCT00396669 | Bupropion |
| NCT02617017, NCT01699828 | Buspirone |
| NCT02542410, NCT00440258, NCT01395602 | Cabergoline |
| NCT03578263 | Carbetocin |
| NCT01212484 | Carbidopa |
| NCT05060549 | Cariprazine |
| NCT03183310 | Chlorpromazine |
| NCT01657760 | Citalopram |
| NCT01684475 | CJH1 (CLR4001) |
| NCT03440112 | Clarithromycin |
| NCT00697814 | Clomiphene citrate |
| NCT00029458 | Clozapine |
| NCT04457310 | CVL-562 (PF-06412562) |
| NCT00000371 | D-cycloserine |
| NCT01116713 | Dexamethasone |
| NCT01296802 | Dexfenfluramine |
| NCT05435560, NCT03170739 | Dexmedetomidine |
| NCT00593138 | Dex-methylphenidate |
| NCT03512171 | Dextroamphetamine |
| NCT00001365 | Dextromethorphan |
| NCT00305331 | Domperidone (drug) |
| NCT02965963, NCT04469959 | Dopamine |
| NCT03714763 | Drug treatment |
| NCT05435729 | DSP-9632P |
| NCT01065558, NCT01215357, NCT01244633, NCT02102698 | Ecopipam |
| NCT00001929 | Eliprodil |
| NCT00801827 | F-18 (fallypride) |
| NCT00592150 | Fenoldopam |
| NCT00603018 | Fluoxetine |
| NCT05318924 | Ghrelin |
| NCT00468806 | GSK598809, using [11C]-(+)-PHNO |
| NCT00814957, NCT01036061 | GSK618334 |
| NCT02203786, NCT00625014 | Haloperidol |
| NCT03305705 | Iron Carboxymaltose |
| NCT02438215 | IRX4204 |
| NCT01140620, NCT05320991 | Ketamine |
| NCT02351128 | Lanreotide |
| NCT00939523 | Lapatinib |
| NCT04588129 | LB-102 |
| NCT00089622 | Levopoda |
| NCT04674670 | Low dose naltrexone |
| NCT03321136, NCT03019822 | LSD |
| NCT02118610 | L-tetrahydropalmatine |
| NCT02333487 | Lu AF35700 |
| NCT01979679 | Lurasidone |
| NCT04968613 | Madopar monotherapy |
| NCT02233556 NCT01108029 NCT03918616 | Memantine |
| NCT02225106, NCT02148783, NCT00015301, NCT02152670, NCT04384562, NCT00301639, NCT00302367, NCT00302393, NCT01924468 | Methylphenidate |
| NCT03560583, NCT02222220, NCT02262923 | Metoclopramide |
| NCT00867360 | Mifepristone (RU-486) |
| NCT01878006 | Morphine |
| NCT01191645 | naloxone |
| NCT02123979 | Neupro® transdermal patch/placebo |
| NCT03999190 | NPA radiotracer |
| NCT00274170 | Octreotide |
| NCT00625170 | Olanzapine |
| NCT03492138, NCT03034200, NCT03099499, NCT05009992 | ONC201 |
| NCT04732065 | ONC206 |
| NCT00934635, NCT00796432 | Paliperidone ER |
| NCT02093169 | Part A: Lu AF35700 |
| NCT01620138 | Pasireotide |
| NCT00595140 | Pegvisomant |
| NCT01066403 | Pergolide |
| NCT02008292 | Physostigmine |
| NCT04373317, NCT03947216 NCT03994965, | Pimavanserin |
| NCT05507372 | Pimozide |
| NCT01519856 | Piribedil (Clarium) |
| NCT02557984, NCT03854942, NCT05405244 | Placebo |
| NCT03970239 | PET with [11 Carbon]-3-amino-4-(2-dimethylaminomethylphenylsulfanyl)-benzonitrile ([11C]-DASB) and [18 Fluorine]-altanserin ([18F]-altanserin) |
| NCT03681509, NCT05355337, NCT00086307, NCT00231959, NCT04121091, NCT00471588 | Pramipexole |
| NCT01999530 | Prazosin Hydrochloride |
| NCT01155466 | Preladenant |
| NCT03019289 | Pridopidine |
| NCT01781377 | Propofol |
| NCT03254186 | Propranolol Hydrochloride |
| NCT00396734 | Provigil |
| NCT01908452 NCT03790345 | Pyridoxine |
| NCT04639960 | Risperdal |
| NCT01510015, NCT01259973, NCT03148509, NCT00825045, NCT02538081, NCT00716755 | Risperidone |
| NCT00335205 | Ropinirole |
| NCT00663338, NCT01723904 | Rotigotine |
| NCT01278342 | Sandostatin LAR |
| NCT00269035 | SB773812 |
| NCT02291861 | SD-809 |
| NCT02579473 | SER-214 |
| NCT00832221 | SEROQUEL XR (quetiapine) |
| NCT05208294 | Sulpiride 400 MG |
| NCT03544229 | TAK-906 Maleate |
| NCT04386317 | Terazosin |
| NCT03059563, NCT01632189, NCT00873535, NCT00713479 | Varenicline |
| NCT02051413 | Venlafaxine extended release |
| NCT02637076 | Xyrem |
| NCT00818298 | Ziprazidone |
| NCT00206960 | Zuclopenthixol |

**Table S2**: Description of interventions classified as “Radiation”

| **StudyID** | **Type of radiation intervention** |
| --- | --- |
| NCT00802204 | PET |
| NCT03911726 | Single PET/MR |
| NCT00670293 | PET using [11C]raclopride |
| NCT01784016 | [11C]-PHNO |
| NCT00302380 | PET using [11C]altropane as the ligand. |
| NCT03334045 | PET/MR |
| NCT05011760 | PET [C-11]NPA |

**Table S3**: Description of interventions classified as “Genetic”

| **StudyID** | **Type of genetic intervention** |
| --- | --- |
| NCT02989792 | Blood sampling for genotyping |
| NCT02897167 | Retrospective study of frozen samples |

**Table S4:** Description of interventions classified as “Diagnostic Test”

| **StudyID** | **Type of diagnostic test** |
| --- | --- |
| NCT04084977 | Antibody detection |
| NCT03537794 | PET scans |
| NCT03485339 | Genome testing |
| NCT05676827 | Diagnostic criteria for temporomandibular disorders clinical examination |
| NCT03815838 | PET imaging |

**Table S5:** Description of interventions classified as “Dietary Supplement”

| **StudyID** | **Type of dietary supplement** |
| --- | --- |
| NCT01094756 | Meal replacements, psychotherapy, dietary education |
| NCT01558193 | Placebo |
| NCT04389723 | Qualia Mind |
| NCT05030129 | 5-Hydroxytryptophan/Vitamin B6 |

**Table S6**: Description of interventions classified as “Device”

| **StudyID** | **Type of device** |
| --- | --- |
| NCT04192058 | Sham transcranial direct current stimulation (tDCS) |
| NCT02455219 | Prototype UHRSZ (UltraHigh Resolution Smart Zoom) Collimator |
| NCT05232955 | PET/MR scanner |
| NCT04086459 | Repetitive transcranial magnetic stimulation |
| NCT02880995 | PET scan |

**Table S7:** Description of interventions classified as “Procedure”

| **StudyID** | **Type of procedure** |
| --- | --- |
| NCT00166322 | [^123^I]-3-Jodo-6-methoxybenzamin-single photon emission computer tomography (IBZM-SPECT, bolus and constant infusion paradigm) |
| NCT02906501 | Penn Web-Based Computerized Neurocognitive Battery |
| NCT03158090 | Transnasal butterfly surgery |
| NCT03715244 | Spinal anesthesia with short-acting local anesthetics |
| NCT01065376 | Timing of cabergoline administration |
| NCT01160991 | Glucose clamp technique |

**Table S8:** Description of interventions classified as “Behavorial”

| **StudyID** | **Type of behavorial intervention** |
| --- | --- |
| NCT03709667 | EX |
| NCT00422500 | Questionnaire |
| NCT02605902 | Comprehensive behavioral intervention for tics |
| NCT04880824 | Prehabilitation- new form of care |

**Table S9:** Description of interventions classified as “Other”

| **StudyID** | **Type of intervention** |
| --- | --- |
| NCT03637075 | Intranasal insulin |
| NCT03317431 | Mechanical ventilation |
| NCT00875407 | Scintigraphy in ^123^I-IBZM |
| NCT02856347 | 18F-DOPA |
| NCT04469283 | Caffeine and movement disorders |
| NCT01142739 | Clinical variables |
| NCT02514720 | Cigarette |
| NCT03037060 | [11C]-(+)-PHNO PET scan |
| NCT02094196 | Alcohol detoxification |
| NCT02706262 | Metabolically abnormal obese – Mediterranean diet |
| NCT03717454 | Surgery |
| NCT04485507 | Nature-VR |
| NCT05359887 | Standardized liquid mixed meal Nutridrink® |
| NCT03998787 | Neurophysiological assessment |
| NCT04893668 | Depression |

**Table S10:** Indications investigated in a single study

| **Trial ID** | **Indication** |
| --- | --- |
| NCT04639960 | 22q11.2 deletion syndrome |
| NCT03317431 | Acute lung injury |
| NCT04469283 | ADCY5-related dyskinesia |
| NCT03334045 | Adjustment disorder with work inhibition |
| NCT01558193 | Aggression |
| NCT00254306 | Amphetamine-related disorders |
| NCT02351128 | Carcinoma, Merkel cell |
| NCT03578263 | Cesarean section complications |
| NCT00875407 | Chromaffin-tissue derived tumors |
| NCT01761032 | Compulsive and infrequent tanners |
| NCT03715244 | Delirium |
| NCT00024960 | Developmental stuttering |
| NCT03637075 | Diabetes mellitus, type 2 |
| NCT00593138 | Drug binding to DAT Receptors |
| NCT03099499 | Endometrial cancer |
| NCT02542410 | Endometriosis |
| NCT03183310 | Esophageal reflux |
| NCT01212484 | Familial dysautonomia |
| NCT05030129 | Fragile X syndrome |
| NCT04880824 | Frailty syndrome |
| NCT00004793 | Growth disorders |
| NCT00592150 | Hypertension |
| NCT01065558 | Lesch-Nyhan syndrome |
| NCT00422500 | Lung cancer |
| NCT02856347 | Medullary thyroid cancer |
| NCT00274170 | Migraine headache |
| NCT03492138 | Multiple myeloma |
| NCT03918616 | Neurodegenerative disease |
| NCT00471588 | Obsessive-compulsive disorder |
| NCT01191645 | Opioid induced pharyngeal and esophageal dysfunction |
| NCT00440258 | Ovarian hyperstimulation syndrome |
| NCT05208294 | Placebo effect on mood improvement |
| NCT02123979 | Post-operative pain |
| NCT01781377 | Pregnancy |
| NCT03717454 | Prolactinoma |
| NCT03170739 | Renal function |
| NCT05507372 | Tinnitus, subjective |
| NCT05676827 | Temporomandibular disorder |
| NCT02148783 | Traumatic brain injury |

**Table S11:** Frequency of studies with results by interventional drug

| Drug | Frequency of studies with results |
| --- | --- |
| [11C]raclopride | 1 |
| Amphetamine | 1 |
| Armodafinil | 1 |
| Brexpiprazole 1mg to 4mg | 1 |
| Cabergoline | 1 |
| Carbidopa | 1 |
| Citalopram | 1 |
| Clarithromycin (Not used as of 4/2020) | 1 |
| D-cycloserine | 1 |
| Ecopipam | 3 |
| F-18 (fallypride) | 1 |
| Fluoxetine | 1 |
| Haloperidol | 1 |
| Lapatinib | 1 |
| L-tetrahydropalmatine (30mg) | 1 |
| Lu AF35700 | 1 |
| Methylphenidate | 2 |
| Mifepristone (RU-486) | 1 |
| Morphine | 1 |
| Paliperidone ER | 1 |
| Pasireotide | 1 |
| Physostigmine | 1 |
| Placebo | 1 |
| Pramipexole | 1 |
| Prazosin Hydrochloride | 1 |
| Preladenant 2 mg tablet | 1 |
| Pridopidine (90 mg) | 1 |
| Rotigotine | 1 |
| Sandostatin LAR | 1 |
| SD-809 | 1 |
| TAK-906 Maleate | 1 |
| Varenicline, then placebo | 1 |
| Xyrem | 1 |

**Table S12:** Preferred Reporting Items for Systematic reviews and Meta-Analyses extension for Scoping Reviews (PRISMA-ScR) Checklist

| **SECTION** | **ITEM** | **PRISMA-ScR CHECKLIST ITEM** | **REPORTED ON PAGE #** |
| --- | --- | --- | --- |
| **TITLE** | | | |
| Title | 1 | Identify the report as a scoping review. | 1 |
| **ABSTRACT** | | | |
| Structured summary | 2 | Provide a structured summary that includes (as applicable): background, objectives, eligibility criteria, sources of evidence, charting methods, results, and conclusions that relate to the review questions and objectives. | 2 |
| **INTRODUCTION** | | | |
| Rationale | 3 | Describe the rationale for the review in the context of what is already known. Explain why the review questions/objectives lend themselves to a scoping review approach. | 5 |
| Objectives | 4 | Provide an explicit statement of the questions and objectives being addressed with reference to their key elements (e.g., population or participants, concepts, and context) or other relevant key elements used to conceptualize the review questions and/or objectives. | 6-7 |
| **METHODS** | | | |
| Protocol and registration | 5 | Indicate whether a review protocol exists; state if and where it can be accessed (e.g., a Web address); and if available, provide registration information, including the registration number. | No protocol registered |
| Eligibility criteria | 6 | Specify characteristics of the sources of evidence used as eligibility criteria (e.g., years considered, language, and publication status), and provide a rationale. | 8 |
| Information sources* | 7 | Describe all information sources in the search (e.g., databases with dates of coverage and contact with authors to identify additional sources), as well as the date the most recent search was executed. | 8 |
| Search | 8 | Present the full electronic search strategy for at least 1 database, including any limits used, such that it could be repeated. | 8 |
| Selection of sources of evidence† | 9 | State the process for selecting sources of evidence (i.e., screening and eligibility) included in the scoping review. | 8 |
| Data charting process‡ | 10 | Describe the methods of charting data from the included sources of evidence (e.g., calibrated forms or forms that have been tested by the team before their use, and whether data charting was done independently or in duplicate) and any processes for obtaining and confirming data from investigators. | 8 |
| Data items | 11 | List and define all variables for which data were sought and any assumptions and simplifications made. | 8-9 |
| Critical appraisal of individual sources of evidence§ | 12 | If done, provide a rationale for conducting a critical appraisal of included sources of evidence; describe the methods used and how this information was used in any data synthesis (if appropriate). | 9 |
| Synthesis of results | 13 | Describe the methods of handling and summarizing the data that were charted. | 9 |
| **RESULTS** | | | |
| Selection of sources of evidence | 14 | Give numbers of sources of evidence screened, assessed for eligibility, and included in the review, with reasons for exclusions at each stage, ideally using a flow diagram. | 10-11 |
| Characteristics of sources of evidence | 15 | For each source of evidence, present characteristics for which data were charted and provide the citations. | 11-38 |
| Critical appraisal within sources of evidence | 16 | If done, present data on critical appraisal of included sources of evidence (see item 12). | 11-38 |
| Results of individual sources of evidence | 17 | For each included source of evidence, present the relevant data that were charted that relate to the review questions and objectives. | 11-38 |
| Synthesis of results | 18 | Summarize and/or present the charting results as they relate to the review questions and objectives. | 11-38 |
| **DISCUSSION** | | | |
| Summary of evidence | 19 | Summarize the main results (including an overview of concepts, themes, and types of evidence available), link to the review questions and objectives, and consider the relevance to key groups. | 11-42 |
| Limitations | 20 | Discuss the limitations of the scoping review process. | 38-40 |
| Conclusions | 21 | Provide a general interpretation of the results with respect to the review questions and objectives, as well as potential implications and/or next steps. | 41 |
| **FUNDING** | | | |
| Funding | 22 | Describe sources of funding for the included sources of evidence, as well as sources of funding for the scoping review. Describe the role of the funders of the scoping review. | 47 |

Figure S1: Location of sponsors in the category “others”


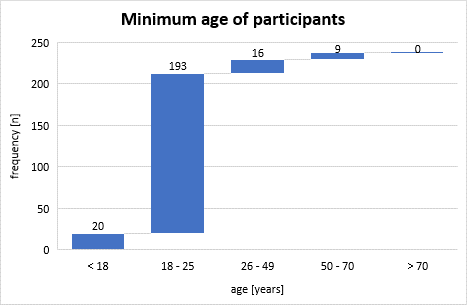


**Figure S2:** Frequency of minimum age categories


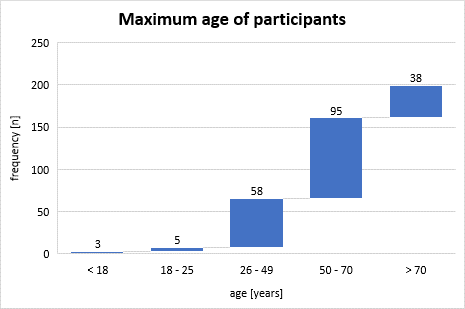


**Figure S3:** Frequency of maximum age categories

**Figure S4:** Study duration for drug interventions

**Figure S5:** Distribution of sponsorship of studies with published results

**FigureS6:** Time trends of studies with and without results

**FigureS7:** Time trends of interventions

Figure S8: Publication trends by indication over time
